# Short-Term Effect of Prismatic Lenses in Head Positioning and Kinematics in Patients with Postural Deficiency Syndrome

**DOI:** 10.3390/healthcare14142120

**Published:** 2026-07-15

**Authors:** João Alves da Silva, Fábio Trindade, Ivo Álvares Furtado, Pedro Fernandes

**Affiliations:** 1 Centro Académico de Medicina de Lisboa, Universidade de Lisboa, 1649-028 Lisbon, Portugal; 2 Faculdade de Ciências da Vida, Universidade da Madeira, 9020-105 Funchal, Portugal; 3 Faculdade de Medicina, Universidade de Lisboa, 1649-028 Lisbon, Portugal; 4 ULS Santa Maria, 1649-035 Lisboa, Portugal

**Keywords:** head movements, range of motion, posture, proprioception, active prisms

## Abstract

Background: Asymmetries in head movement are common in postural disturbances. The correction of such changes using prismatic lenses has been reported but not quantified. Methods: A controlled randomized double-blind prospective study was performed on a population with Postural Deficiency Syndrome, comparing active head extension and rotation using prismatic and sham lenses. The evaluation was carried out using clinical photography and goniometer before intervention and 15 min after lenses’ application. Results: One hundred patients were included. The initial extension from a horizontal gaze was 53.2±12.6°, and the rotation amplitude was 113±15.2°. The overall head movement was below the standard reference values of 75° for head extension and 160° for rotation. (p<0.01 for both variables). The intervention group exhibited both an extension increase and rotation increase in amplitude: rotation improved by 8.7±9.9° (p<0.01), and extension increased on average 6.5±8.0° (p<0.01). The initial head rotation asymmetry was 10.1±4.3°, reducing to 1.70±1.58° after intervention (p<0.01). Conclusions: Patients with Postural Deficiency Syndrome have a limited and asymmetrical range of motion of head rotation and extension. These limitations are responsive to the use of low-powered prismatic lenses.

## 1. Introduction

The coordination of head and neck movement is very complex both mechanically and neurologically. The eight bone segments and their twenty-three joints must be harmonically mobilized by multiple segmental muscle layers so that their positioning maintains function, balance and direction of gaze. Therefore, its musculoskeletal proprioception must coordinate with vestibular, retinal and eye muscle inputs. Such interactions and adjustments are evident in laboratory settings [1,2]. A complex system such as this can frequently become dysfunctional, with patients presenting a reduced range of motion, pain and changes in sensorimotor control without structural abnormality [3].

The Postural Deficiency Syndrome (PDS) was first described by physiatrist Henrique Martins da Cunha as a complex clinical presentation secondary to a patient’s deviation from the ideal biomechanics and a deficiency of proprioceptive information [4]. The functional asymmetrical positioning of the head and neck is a cardinal symptom of PDS, as proprioceptive errors limit the ability to coordinate and relax the distinct agonist–antagonist muscles encompassing head extension and rotation [5]. Despite the polymorphic presentation of PDS, postural asymmetry is a constant sign and is used for its diagnosis and classification [6].

The evaluation of static head and neck asymmetrical positioning by Martins da Cunha and later dynamical identification of extension and rotation disparities by Alves da Silva were only qualitatively published [4,5,6], and published peer-reviewed literature on this subject is scarce. The subjective descriptions of the syndrome were based on clinical observations reporting clear deviations of head and neck static and dynamics, but no objective, clinically meaningful measurements have been published to our knowledge. These inaccuracies pose a significant clinical gap for both the identification of PDS and its clinical follow-up.

The complexity of this segment’s anatomy and physiology makes its accurate evaluation difficult and multiple techniques have been applied to obtain precise and reliable measurements of cervical motion [7]. Goniometers, inclinometers and measuring tape are accessible in the simplest settings and have high reliability, with most tested equipment with intraclass correlation coefficients for intra and interobserver exceeding 0.85 and 0.70, respectively [8]. Imaging such as clinical photography and X-rays have been used for almost a century [9]. They are precise and may address dynamics [10,11], but its biplanar limitation cannot encompass the complex three-dimensional components of neck motion. CT and MRI perform three-dimensional acquisitions but struggle to replicate common motion and positioning. The most accurate measurements of cervical motion include optoelectronic scanners with 3D computerized acquisitions. However, these tools are expensive, cumbersome and complex to interpret, making them impractical in a clinical setting [10].

The treatment of proprioceptive disturbances in PDS may include the use of low-powered prismatic lenses (LPPLs) oriented according to the oculomotor muscles’ axis [6,12]: deviations of the retinal image position up to 4 diopters are compensated by a tonic adjustment in those muscles [13], and therefore, the use of LPPL in order to provoke such changes has a direct reflex action on the proprioceptive system of the head and neck positioning muscles [1,2,14,15]. These changes occur in two different neurologic pathways [16]: a fast recalibration pathway reflects the strategic adjustment of spatially coded movement commands aimed at rapidly reducing reaching errors. This optokynetic response has a latency of 160–200 milliseconds in healthy humans, and it is an occipito-temporal and inferior parietal cortex process. A distinct pathway of realignment is a slower process of progressive remapping between visual and proprioceptive coordinate frames and is evidenced after 3 to 5 min in functional MRI (fMRI) as parieto-cerebellar activity [17].

The research question of this study is: Can the use of low-powered supero-external base prisms alter axial mobility in patients with PDS? We hypothesize that the application of LPPLs will significantly improve cervical axial mobility by correcting head positioning asymmetries and optimizing cervical kinematics in PDS patients.

## 2. Materials and Methods

### 2.1. Study Design and Rationale

A controlled prospective double-blind randomized study was designed. The objective of this study is to quantify the head movement limitation and asymmetry present in patients with PDS in a clinical setting, and its short-term response to the use of LPPLs. As the syndrome is described as presenting “clear deviations” [4] of head and neck positioning, the study was designed to use measurement tools that balance precision, ease of use and reproducibility in a clinical setting. Two central parameters of PDS were chosen for evaluation: head extension and head rotation, for these are described as the reference findings for the application of the prism prescription protocol in clinical practice [6]. A fifteen-minute interval between observations was decided according to the known timeframe of postural adaptation to prismatic lenses in healthy subjects [16,17]: the extremely fast optokynetic reflex is a “fast and forgetful” neural circuit, while the slower postural recalibration circuit presents “slow and stable learning” characteristics [18].

Three primary outcomes were defined from the design: a change in the amplitude of head extension, a change in the amplitude of head rotation and a change in the asymmetry of head rotation.

### 2.2. Ethical Approval Declarations

This investigation was designed in accordance with the Declaration of Helsinki and European guidelines for clinical trials in humans. It was submitted to and approved by the ethics committee of the author’s university. All patients signed an informed consent.

### 2.3. Participants and Sample-Size Calculations

Subjects were recruited from two clinical centers, and those meeting the eligibility criteria were included in the study. The authors were unable to find any published objective measurements of the study’s parameters. Given the absence of robust data in which to base the sample size estimation required to achieve the desired alpha level, a pilot group of 12 patients was observed to provide a basis for estimation [19]. The three primary outcomes were recorded, and a sample size for two averages was estimated for these variables using Cohen’s d calculation, statistical power of 0.90 and a significance level of 0.01. The largest sample estimation size calculated predicted *n* = 36.8 observations for each group. A sample size of 100 participants was chosen.

### 2.4. Eligibility Criteria

Inclusion criteria were defined as presentation of diagnostic criteria for PDS comprising (I) two or more cardinal signs of PDS, as defined by Martins da Cunha and detailed in Table A1 and Table A2 in Appendix A; (II) clinical presentation of asymmetrical stance and head extension and rotation; (III) presence of typical directional pseudoscotoma described in this syndrome [6].

Exclusion criteria consisted of (I) vision under 8/10, strabismus or nistagmus; (II) previous eye or vestibular surgery; (III) known disease affecting the central nervous system or inner ear; (IV) medication with known interference with balance and posture and (V) treatment for postural and movement disorders in the previous year.

### 2.5. Patient Randomization

Patients were randomized according to a predefined randomization sequence extracted from www.random.org. This sequence was generated before the recruitment phase and was only known by an ophthalmology specialist responsible for the clinical diagnosis. This specialist did not participate in the remaining procedures, data processing or manuscript writing.

### 2.6. Experimental Procedures

Patients were initially evaluated by a senior ophthalmology specialist, responsible for the diagnosis and refractive correction as necessary. Patients were then evaluated in a distinct office by the first author. After the initial observation, patients would return to the initial office, and either prisms or sham lenses were added by the ophthalmologist, according to the randomization sequence. A second evaluation was performed by the author 15 min later. All visual and study lenses were applied using a standard optical trial frame and lenses (Shin-Nippon TL-34P trial lens set and TF-3 Trial Frame, Rexxam Co., Ltd., Osaka, Japan).

Intervention lenses were applied according to the standard prescription criteria of PDS: prismatic power used is always different for right and left eyes. The vast majority of cases used three and two diopters with prisms at 125° on the right eye and 55° on the left eye, with the highest power applied on the side where head rotation was most limited [6]. The protocol for prism prescription is presented in Table A3 in Appendix A.

Head rotation was measured as the amplitude from the maximal left and right rotation in regard to the mentum and the biacromial line. A 50 cm wide semicircular protractor with one-degree steps was used (WISSNER GmbH, Bensheim, Germany).

**Figure 1:**
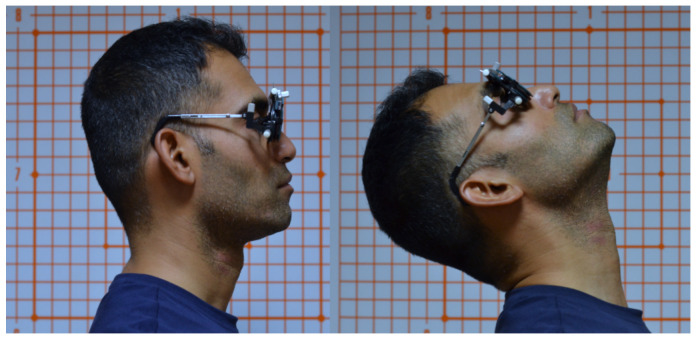
Example of photographic aquisition (not a study subject).

Head extension was measured using clinical photography as the amplitude from horizontal gaze to maximal extension, using the tragus-subnasal reference line. Lateral-view photographs in front of a graduated and ground-leveled Weiss screen [20] were performed using a digital camera on a tripod at 2 m distance (Nikon D5100 with Nikkor 50 mm f/1.8 AF-D lens, Nikon Corporation, Tokyo, Japan). An acquisition example is presented in Figure 1.

### 2.7. Masking

Neither the participant nor the examiner performing the measurements was aware of the intervention assignment. The diagnosis and randomization sequence were only available to the ophthalmology specialist. The observations were performed in a separate room. The senior specialist was not present during either evaluation.

**Figure 2:**
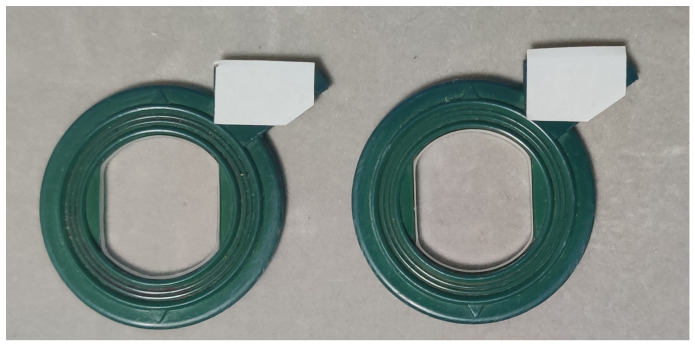
Tagged lenses: sham (**left**) and 2-diopter prism (**right**).

**Figure 3:**
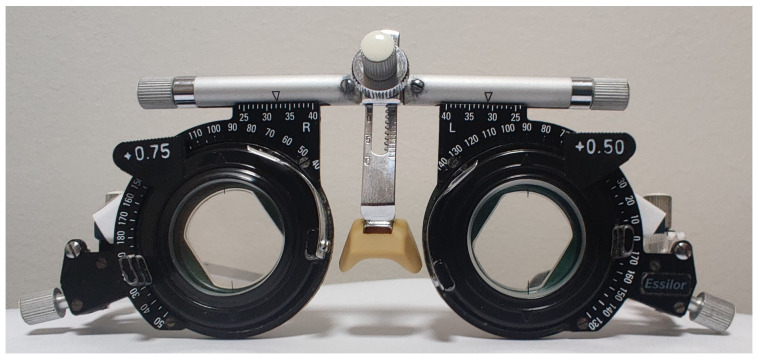
Optical frame with prescription and study lenses.

To prevent potential examiner unmasking by observation of the glasses, the sham and LPPLs were equally tagged in order to make them indistinguishable for both the patient and the author responsible for the measurements, as shown in Figure 2. For further concealment, these lenses were placed in the posterior segment of the trial frame by the ophthalmologist, as presented in Figure 3. No handling of the frame or lenses was performed by the observer. These masking procedures were based on previous publications regarding prism-related prospective randomised double-blind trials [21].

### 2.8. Statistical Analysis

All statistical analyses were performed using R Statistical Software v4.3.3 [22]. Descriptive statistics were performed according to Tukey’s exploratory data analysis principles. The control and intervention groups’ values were evaluated for normality and variance using the Shapiro–Wilk test and the F test. Cohen’s d test was used for the sample-size estimate and the primary outcomes’ effect size. A one-way analysis of covariance (ANCOVA) was used to evaluate covariance by age and gender. A Two Sample *t*-test, a Welch Two Sample *t*-test and a Wilcoxon rank sum test with continuity correction were used accordingly for group comparisons in Table 1 and Table 2. The Wilcoxon signed-rank test was used for paired observations in Figure 6. No subgroup analysis was performed.

## 3. Results

**Figure 4:**
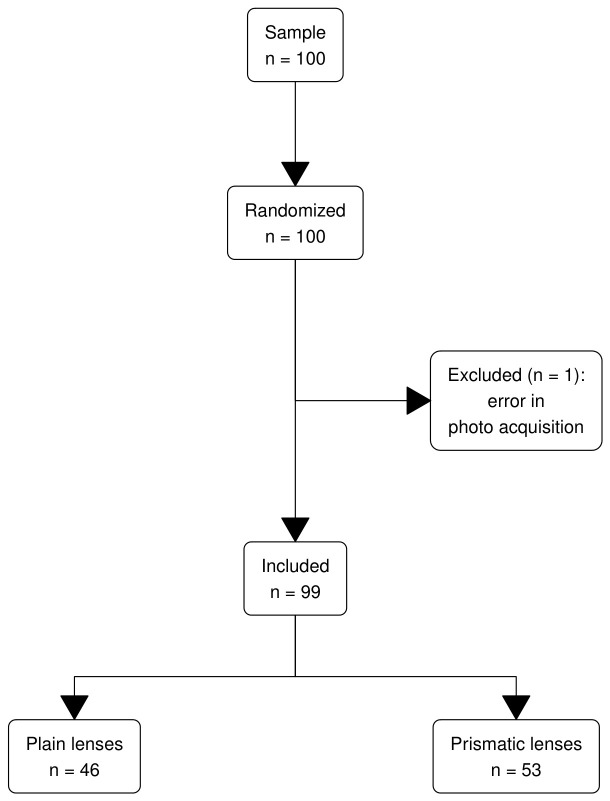
Study flowchart.

A total of 100 consecutive patients were included in the study, as presented in the flowchart (Figure 4). One patient was excluded from the per protocol set due to missing data, secondary to technical error in clinical photo acquisition: a camera setting error resulted in inadequately short exposition time rendering the pictures unmeasurable. After breaking the blind, it was verified that this patient would have been assigned to the intervention group. Forty-six patients were randomized into the control group, and 53 were in the intervention group.

The control and intervention groups had no statistically significant differences at the base parameters, as shown in Table 1. The average maximal extension from horizontal gaze was 53.2±12.6°, and the average rotation amplitude was 113±15.2°. Head extension and rotation presented a moderate negative correlation with age, with r=−0.45 and −0.40, respectively (p<0.01 in both variables). Taking into account normal reference values for head extension and rotation as 75° and 160°, respectively [23], the amplitude was reduced both in extension and in rotation (p<0.001 in one-sample *t*-test for both variables). Patients presented asymmetrical left–right rotation, with a mean difference of 10.1±4.3°.

The intervention and control groups presented statistically relevant changes regarding head rotation and extension, both in amplitude and asymmetry.

Head extension increased by an average 6.5±8.0° in the intervention group, comparing to −0.6±7.1 in the control group. This change was statistically significant between groups in a global evaluation (p<0.01 in *t*-test, 95% CI 10.07 to 4.03) and after adjusting for age (F(1,94)=18.89, p<0.01). The effect size, as measured by Cohen’s d, was d = 0.95, indicating a large effect.

**Figure 5:**
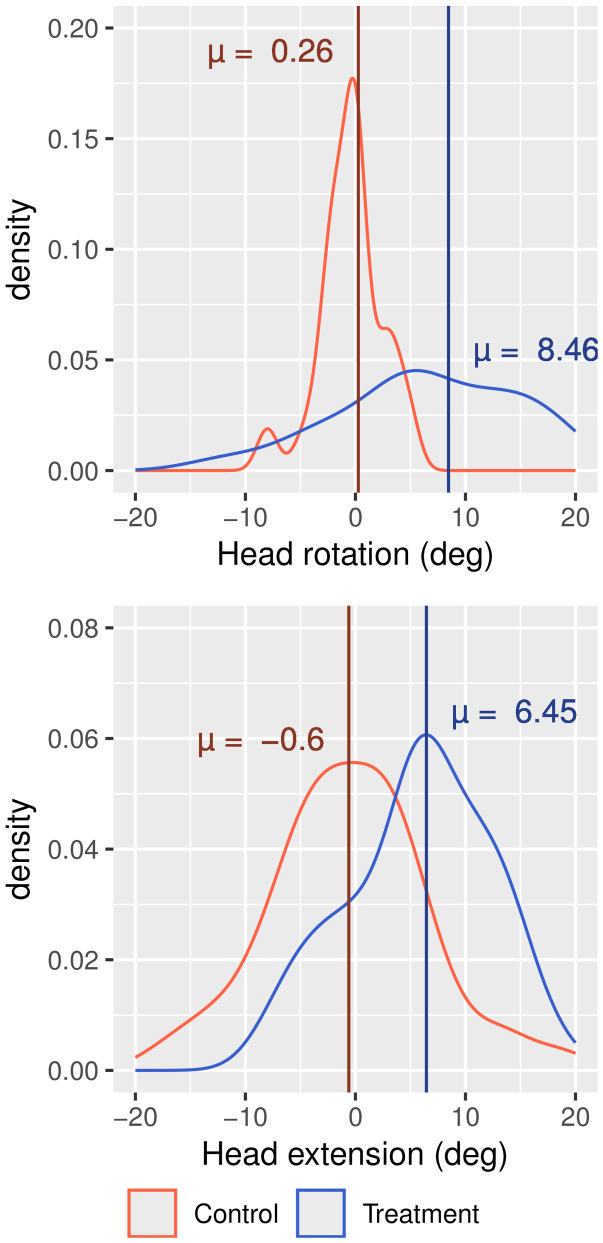
Changes in head rotation and extension.

Intervention group rotation increased by an average of 8.7±9.9° versus 0.3±5.3° in the control group, a statistically significant change globally (p<0.01 in *t*-test, 95% CI 14.81 to 3.54) and after adjusting for age (F(1,94)=32.99, p<0.01). Cohen’s d value was 1.01, indicating a large effect between groups. These changes are presented in Figure 5.

**Figure 6:**
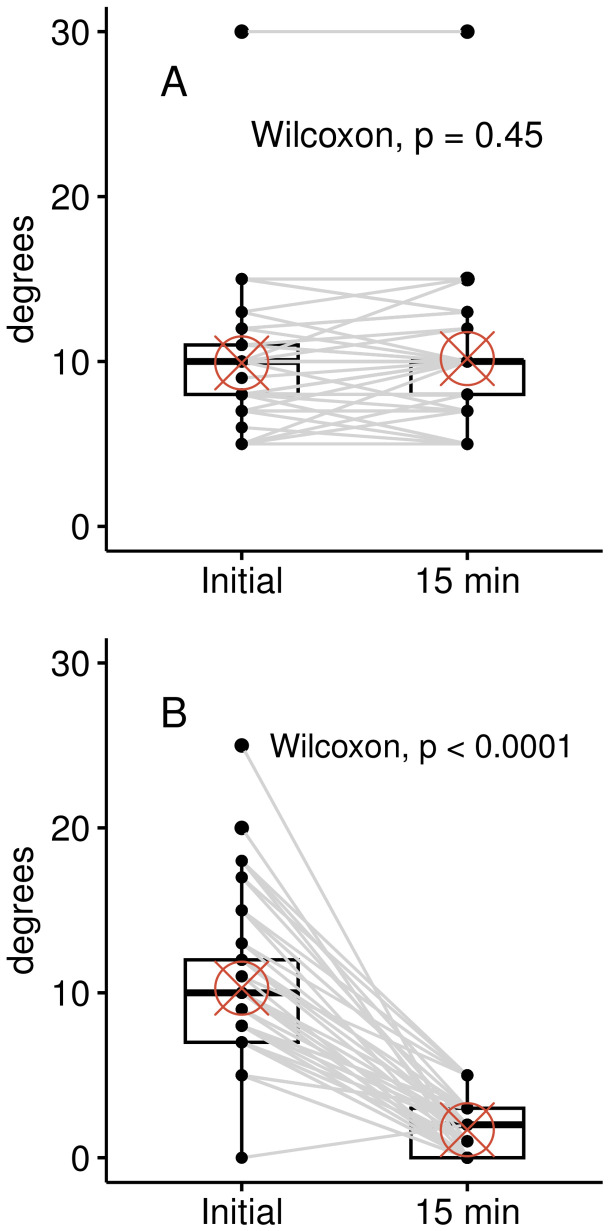
Head rotation asymmetry by intervention group. (**A**): control; (**B**): prisms.

The mean head rotation asymmetry was decreased after the intervention, from 10.3° to 1.7±1.6°, compared to no relevant change in the control group (*p* = 0.45). This change was statistically significant between groups in global evaluation (p<0.01, 95% CI 7.00 to 9.00 in Wilcoxon rank sum test with continuity correction), and after adjusting for age (F(1,94)=315.70, p<0.01). Cohen’s d value was 2.48, indicating a large effect between groups. These changes are expressed in Figure 6. Global values after intervention are reported in Table 2.

## 4. Discussion

The first objective of this study was to clarify and quantify the initial statement that patients with PDS had limited and asymmetrical head positioning as a consequence of proprioceptive dysfunction. The results of this investigation demonstrate that patients with PDS do present restrictions in head and neck movement, with considerable deviations from reference values of head extension and rotation: the mean rotation amplitude is reduced by over 45°, and the mean extension amplitude is restricted up to 20°. The head rotation asymmetry averages 10°.

The second objective of this study was to evaluate the short-term response of head and neck movement to the use of LPPLs in these patients. The group assigned to the prism intervention shows an improvement in all three parameters compared to the control group. Despite the absence of significant asymmetry, extension and rotation remain inferior to reference literature.

The magnitudes of change—over 5°—can be perceived in clinical examination and measurement with easy access tools such as a goniometer and clinical photography [7,8,10].

Studies of the cervical postural effect of oculomotor muscle tone manipulation in humans have so far been limited to healthy volunteers in laboratory settings. Mapping of the reflex correlation between specific oculomotor muscles and axial musculature has been performed: as sensory input from muscle spindles is artificially modified, the spatial coding of visual and skeletal proprioception is recalibrated, with postural and kinesthetic adaptations [1,15,24,25,26].

The effect of prisms in sensorimotor plasticity has been studied for over a century, as prism-induced changes in visual location produce an adaptation of motor behavior for efficient mobility [27]. The use of prisms to correct postural information has been established for 40 years [12,14]. LPPLs are used in postural disturbances to induce similar tonic changes in oculomotor muscles evidenced in known laboratory experiments. Like in the laboratory settings, asymmetrical LPPLs evoke tonic adaptation of the axial muscles and recalibrate the elaboration of body references [6]. Despite the prolonged use of LPPLs in multiple proprioceptive disturbances [6,28], their impact on cervical mobility has not been quantified in patients with postural disturbances.

Proprioceptive dysfunction has been evidenced in patients with neck pain [3,29], and motor proprioceptive acuity reduction impacts mobility and even cognitive functions [30,31].

The neural mechanisms of visual and motor adaptation to prismatic lenses have been studied clinically and using fMRI, and at least two neural pathways have been identified in the initial response, with increased brain activity. Motor recalibration is perceived within minutes, as the proprioceptive system adapts to the distorted visual information [16,17]. A distinct process of brain neuroadaptation has been evidenced months after visual changes secondary to intraocular lenses, with brain activity regularization toward a non-effort pattern six months after surgery [32], but to our knowledge, no similar studies were performed using prismatic lenses. This study measures the effect of LPPL on head and neck mobility within the reflex and calibration responses. This information on the initial adaptation of head and neck mobility in patients with PDS may provide a basis for future investigations on the durability of these changes and eventually the medium and long-term effects of LPPL treatment.

### Study Limitations

This study was carried out at a single center with a focus on the prismatic treatment of PDS. Since PDS has a wide variability in presentation and treatment options, our sample may not accurately represent all patients with PDS due to referral bias.

This study focused exclusively on evaluating the short-term movement amplitude of PDS patients, as it sought to establish measurable characteristics at presentation and short-term variation with prisms. As no treatment endpoints were investigated, this methodology cannot establish any clinical significance apart from the increase in rotation and extension amplitude. Further studies with distinct primary goals and time frames are required to access the clinical value of low-powered prismatic treatment in PDS.

## 5. Conclusions

Patients with PDS present head and neck mobility restrictions, with constrained extension and rotation amplitude.

The range of motion in rotation is asymmetric, with a left-right mismatch averaging 10°.

These limitations in movement amplitude are reduced at 15 min when using LPPL.

The rotation symmetry is restored at 15 min when using LPPL.
**Key findings**Cervical extension is limited in PDSCervical rotation is limited and asymmetrical in PDSLPPL usage increases cervical extension and rotation in PDS over the short termLPPL usage restores rotation symmetry in PDS over the short term.

### Future Research Directions

This study sought to identify objective and measurable basic characteristics of PDS patients at presentation and their short-term responses to LPPL. Its base values may allow the design of further studies seeking to evaluate the criteria for the diagnosis of PDS and the medium- and long-term effects of the usage of prisms in both patients’ mobility and clinical signs. The exact mechanisms of LPPL postural adaptation of the head and neck are not clarified in this study, and further studies allowing precise segmental evaluation, such as dynamic radiography or 3D computerized scanning may allow a better understanding of the motor adaptation of postural disturbances to prismatic lenses.

## Figures and Tables

**Table 1:** Base values of patients by randomization.

	Total (n = 99)	Control (n = 46)	Prisms (n = 53)	*p*
**Gender**				
Female	70 (70.7%)	37 (80.4%)	33 (62.3%)	0.08
Male	29 (29.3%)	9 (19.6%)	20 (37.7%)	
**Age (years)**				
Mean (SD)	39.5 (17.5)	42.3 (18.2)	37.0 (16.5)	0.12
Median [Min, Max]	42.0 [8.00, 76.0]	44.5 [8.00, 76.0]	40.0 [8.00, 72.0]	
**Head extension (deg)**				
Mean (SD)	53.2 (12.6)	54.1 (13.2)	52.5 (12.2)	0.54
Median [Min, Max]	54.5 [19.9, 89.5]	55.0 [22.5, 89.5]	52.7 [19.9, 76.6]	
**Head rotation (deg)**				
Mean (SD)	113 (15.2)	113 (16.6)	113 (14.1)	0.80
Median [Min, Max]	110 [80.0, 151]	114 [80.0, 151]	109 [85.0, 143]	
**Rotation asymmetry (deg)**				
Mean (SD)	10.1 (4.32)	9.91 (4.03)	10.3 (4.59)	0.88
Median [Min, Max]	10.0 [0, 30.0]	10.0 [5.00, 30.0]	10.0 [0, 25.0]	

**Table 2:** Results at intervention endpoint.

	Control (n = 46)	Prisms (n = 53)	*p*
**Head extension**			
Mean (SD)	53.5 (12.0)	59.0 (12.5)	0.03
Median [Min, Max]	52.7 [30.6, 84.6]	58.0 [23.7, 87.1]	
**Change in head extension**			
Mean (SD)	−0.596 (7.13)	6.45 (8.00)	<0.01
Median [Min, Max]	−0.940 [−17.3, 18.7]	5.97 [−7.88, 41.3]	
**Head rotation**			
Mean (SD)	113 (15.7)	122 (12.5)	<0.01
Median [Min, Max]	112 [85.0, 150]	120 [100, 148]	
**Change in head rotation**			
Mean (SD)	0.261 (5.28)	8.66 (9.86)	<0.01
Median [Min, Max]	0 [−8.00, 30.0]	8.00 [−13.0, 37.0]	
**Rotation asymmetry**			
Mean (SD)	10.2 (4.02)	1.70 (1.58)	<0.01
Median [Min, Max]	10.0 [5.00, 30.0]	2.00 [0, 5.00]	
**Change in rotation asymmetry**			
Mean (SD)	−0.261 (1.87)	8.58 (4.57)	<0.01
Median [Min, Max]	0 [−5.00, 3.00]	8.00 [−2.00, 25.0]	

All values in degrees.

## Data Availability

The study protocol and anonymized individual participant data that underlie the results reported in this article may be accessed in the Supplementary Materials. Clinical photography or any other information that may lead to patient’s identification will not be shared.

## Supplementary Materials

The following supporting information can be downloaded at: https://www.mdpi.com/article/10.3390/healthcare1010000/s1, anonymised patient data.

## Abbreviations

The following abbreviations are used in this manuscript:

PDS	Postural Deficiency Syndrome
LPPL	low-powered prismatic lenses
fMRI	functional magnetic resonance imaging

## Appendix A. Clinical Criteria for the Diagnosis of PDS

This appendix replicates the original publication of PDS functional and cardinal signs from Martins da Cunha [5] and the prism prescription protocol [6] used in this paper.

**Table A1:** Original definition of functional signs of PDS [5].

Signs	Clinical Presentation
Pain	Headache, retro-ocular pain, thoracic or abdominal, articular, axial
Imbalance	Nausea, dizziness, vertigo, unexplained falls
Ophtalmologic signs	Asthenopia, deranged vision, diplopia, direccional scotoma, metatopsia
Signs of proprioceptive nature	Dysmetria, somatoagnosia, errors in appreciation of the body schema

**Table A2:** Original definition of cardinal signs of PDS [5].

Signs	Clinical Presentation
Articular	Temporomandibular syndrome, torticolis, lumbago, periarthritis, sprains
Neuromuscular	Paresis, loss of motor control of limbs
Neurovascular	limb paresthesias, Raynaud’s phenomenon
Cardio-circulatory	Tachycardia, lipotimia
Respiratory	Dyspnea, fatigue
Otorhinolaryngologic	Buzzing, deafness
Psychic	Dyslexia, dysgraphia, agoraphobia, lack of orientation, spatial and right–left localization errors.
	Concentration deficit, loss of memory, asthenia, anxiety, depression.

**Table A3:** Prism prescription protocol in PDS [6].

PDS Type	Prism Prescription
Pure right	RE 180° 3Δ
Pure left	LE 0° 3Δ
Mixed predominant right	RE 125° 2 or 3Δ
Mixed predominant left	LE 55° 2 or 3Δ
Mixed pure right	RE 125° 3Δ and LE 55° 2Δ
Mixed pure left	RE 125° 2Δ and LE 55° 3Δ
